# Insulin Resistance and Cancer Risk: An Overview of the Pathogenetic Mechanisms

**DOI:** 10.1155/2012/789174

**Published:** 2012-06-04

**Authors:** Biagio Arcidiacono, Stefania Iiritano, Aurora Nocera, Katiuscia Possidente, Maria T. Nevolo, Valeria Ventura, Daniela Foti, Eusebio Chiefari, Antonio Brunetti

**Affiliations:** ^1^Department of Health Sciences, Magna Græcia University of Catanzaro, Viale Europa (Località Germaneto), 88100 Catanzaro, Italy; ^2^Clinical Pathology, Magna Græcia University of Catanzaro, Viale Europa (Località Germaneto), 88100 Catanzaro, Italy; ^3^Endocrinology, Magna Græcia University of Catanzaro, Viale Europa (Località Germaneto), 88100 Catanzaro, Italy

## Abstract

Insulin resistance is common in individuals with obesity or type 2 diabetes (T2D), in which circulating insulin levels are frequently increased. Recent epidemiological and clinical evidence points to a link between insulin resistance and cancer. The mechanisms for this association are unknown, but hyperinsulinaemia (a hallmark of insulin resistance) and the increase in bioavailable insulin-like growth factor I (IGF-I) appear to have a role in tumor initiation and progression in insulin-resistant patients. Insulin and IGF-I inhibit the hepatic synthesis of sex-hormone binding globulin (SHBG), whereas both hormones stimulate the ovarian synthesis of sex steroids, whose effects, in breast epithelium and endometrium, can promote cellular proliferation and inhibit apoptosis. Furthermore, an increased risk of cancer among insulin-resistant patients can be due to overproduction of reactive oxygen species (ROS) that can damage DNA contributing to mutagenesis and carcinogenesis. On the other hand, it is possible that the abundance of inflammatory cells in adipose tissue of obese and diabetic patients may promote systemic inflammation which can result in a protumorigenic environment. Here, we summarize recent progress on insulin resistance and cancer, focusing on various implicated mechanisms that have been described recently, and discuss how these mechanisms may contribute to cancer initiation and progression.

## 1. Introduction/General Overview

**Figure 1:**
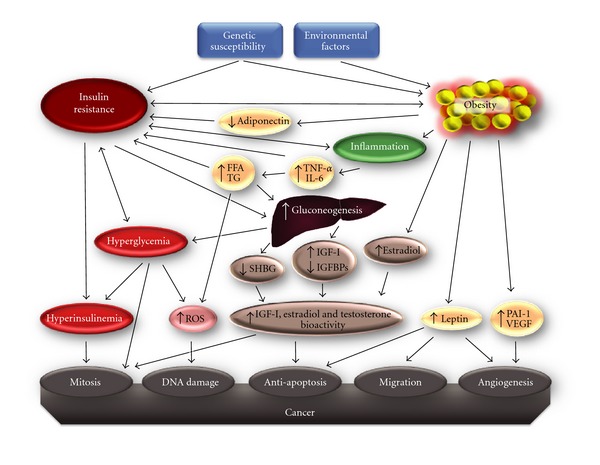
A multidimensional model of cancer development, which suggests insulin resistance and inflammation as driving forces behind cancer. TG: triglycerides; FFA: free fatty acids; TNF-*α*: tumor necrosis factor *α*; IL-6: interleukin-6; ROS: reactive oxygen species; SHBG: sex-hormone-binding globulin; IGF-I: insulin-like growth factor I; PAI-1: plasminogen activator inhibitor-1; IGFBPs IGF-I binding proteins; VEGF, vascular endothelial growth factor.

Insulin resistance is a pathological condition in which insulin action is impaired in peripheral target tissues including skeletal muscle, liver, and adipose tissue. Initially, in individuals destined to develop T2D, the pancreatic beta cells increase insulin production to overcome insulin resistance and maintain euglycemia. Frank T2D in insulin-resistant individuals develops when beta cells fail to compensate [1, 2]. Also, insulin resistance is a cardinal feature of the metabolic syndrome, a quartet of vascular risk factors which include, in addition to insulin resistance, central obesity, dyslipidemia, and systemic hypertension [3]. With the exception of rare, monogenic forms of insulin resistance, common insulin resistance is a very heterogeneous disorder for which both genetic and environmental factors jointly determine susceptibility [4]. The environmental component reflects the unfavorable global shift toward a western lifestyle of overeating and sedentary habits, with obesity as the outcome [2, 5]. The genetic factor is linked to quantitative and/or qualitative defects in the insulin receptor (INSR) signaling pathway which regulates growth and metabolic responses to insulin, in insulin target cells and tissues [6]. Patients with insulin resistance show an increased morbidity and mortality, largely attributable to cardiovascular disease and T2D [7, 8]. Moreover, a number of epidemiological studies have consistently demonstrated that the risk for several types of cancer (including that of the breast, colorectum, liver, and pancreas) is higher in insulin-resistant patients [9]. As illustrated in Figure 1, various mechanisms have been proposed to explain this link, although a complete picture is yet to emerge. The following is a summary of major specific issues currently under debate, related to this area of research.

1. Chronic hyperinsulinemia, in affected individuals, may promote cancer, as insulin can exert its oncogenic potential via abnormal stimulation of multiple cellular signaling cascades, enhancing growth factor-dependent cell proliferation and/or by directly affecting cell metabolism.
2. Insulin increases the bioactivity of IGF-I by enhancing hepatic IGF-I synthesis and by reducing hepatic protein production of the insulin-like growth factor binding proteins 1 (IGFBP-1) and 2 (IGFBP-2) [10, 11]. Therefore, although insulin can directly induce tumour growth, many of its mitogenic and antiapoptotic effects are operating through the IGF-I system, as reported in individuals with high levels of circulating IGF-I, in which an increased risk of developing certain types of tumours, in particular breast and prostate cancers, has been documented [12, 13].
3. Insulin, by reducing SHBG levels, exerts a positive effect on estrogen bioavailability, therefore increasing breast cancer risk.
4. Obesity, the most common cause of insulin resistance, is increasingly recognized as a low-grade inflammatory state in which overproduction of certain molecules, such as free fatty acids, interleukin-6 (IL-6), adiponectin, leptin, tumour necrosis factor alpha (TNF-*α*), plasminogen activator inhibitor-1, and monocyte chemoattractant protein (MCP-1), can play a role in malignant transformation and/or cancer progression [14]. In this context, chronic hyperglycemia and increased oxidative stress may also contribute to increased cancer risk.

Therefore, many lines of evidence support the concept that a relationship exists between insulin resistance and cancer, although further studies must be done before this relationship can be fully understood.

## 2. The INSR, Biological Function, and Its Clinical Significance in Cancer

**Figure 2:**
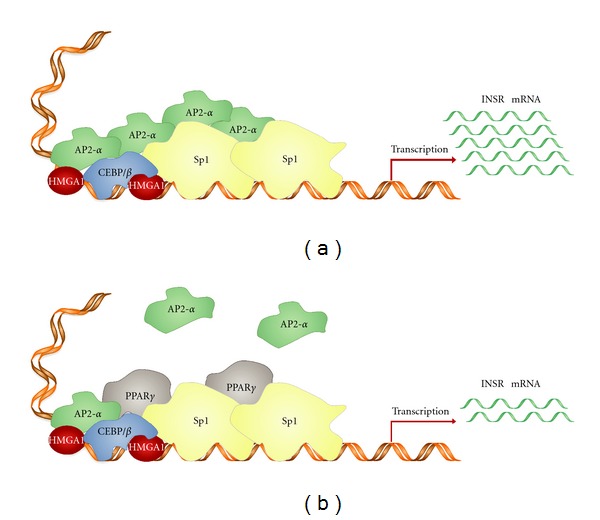
*INSR* gene expression in breast cancer. (a) AP2-*α* overexpression increases INSR expression in breast tumour [37]. Transactivation of the *INSR* gene by AP2-*α* occurs indirectly through physical and functional cooperation with HMGA1 and Sp1. (b) By binding to AP2-*α* and Sp1, PPAR*γ* and agonists may attenuate the stimulatory effect of AP2-*α* on *INSR* gene transcription in breast cancer.

The first step in insulin action is its interaction with the INSR, an integral membrane glycoprotein with intrinsic enzymatic activity. The INSR belongs to the tyrosine kinase growth factor receptor family and functions as an enzyme that transfers phosphate groups from ATP to tyrosine residues on intracellular target proteins, thus playing a critical role in both directing the hormone to a specific target tissue and programming the biological response of the tissue to the hormone. The INSR consists of two identical extracellular *α* subunits (130 kDa) that house insulin binding domains and two transmembrane *β* subunits (95 kDa) that contain ligand-activated tyrosine kinase activity in their intracellular domains [15–18]. Upon binding of insulin to the *α* subunits, the receptor becomes activated by tyrosine autophosphorylation, and then the INSR tyrosine kinase phosphorylates various intracellular effector molecules (e.g., IRS proteins and Shc) which in turn alter their activity, thereby generating a biological response [16–19]. The INSR exists as two splice variant isoforms: the INSR-B isoform that is responsible for signaling metabolic responses involved mainly in the regulation of glucose uptake and metabolism and the INSR-A isoform that is expressed in certain tumours (such as mammary cancers), signals predominantly mitogenic responses, and is capable of binding IGF-II with high affinity [20, 21]. As a consequence of these cellular activities, abnormalities of INSR expression and/or function can facilitate the development of several metabolic and neoplastic disorders. Abnormalities in the INSR signaling pathway are implicated in certain common dysmetabolic disorders, including obesity, T2D, the metabolic syndrome, and the polycystic ovary syndrome [22–25]. Also, rare clinical syndromes due to mutations in the *INSR* gene have been identified in patients with monogenic forms of severe insulin resistance [26, 27]. A relation between INSR and cancer has been established following the observation that overexpression of functional INSRs can occur in human breast cancer and other epithelial tumours, including ovarian and colon cancer, in which the INSR may exert its oncogenic potential via abnormal stimulation of multiple cellular signaling cascades, enhancing growth factor-dependent proliferation, and/or by directly affecting cell metabolism [28–33]. On the other hand, epidemiological and clinical evidence points to a link between insulin-resistant syndromes, such as obesity and T2D, and cancer of the colon, liver, pancreas, breast and endometrium. The mechanistic link between insulin resistance and cancer is unknown, but constitutive activation of the tyrosine kinase activity of INSR and related downstream signaling pathways by chronic sustained hyperinsulinemia, in these clinical syndromes, appears to have a role in the neoplastic transformation process [34–36]. Mechanisms due to hyperinsulinemia that promote malignancy and neoplastic progression include the increase in IGF-I and sex hormones bioavailability, the increase in proinflammatory cytokines, and oxidative stress. Although the molecular mechanisms that cause neoplastic transformation, and sustain tumour progression in the presence of INSR hyperexpression and/or hyperstimulation are not fully understood, an explanation for increased INSR expression in epithelial tumours has been recently provided by our group in both breast cancer cell lines and human breast cancer tissues, in which overexpression of the nuclear transcription factor activator protein 2-*α* (AP2-*α*) accounted for INSR overexpression [37] (Figure 2(a)). In these cases, we demonstrated that transactivation of the *INSR* gene by AP2-*α* represented a fundamental prerequisite to activate *INSR* gene transcription in neoplastic breast tissue. Similarly, a functional link between INSR and cyclin D1 has been recently described in pancreatic cancer [38]. Thiazolidinediones (TZDs), a class of commonly used antidiabetic drugs that act as peroxisome proliferator-activated receptor (PPAR*γ*) agonists, have shown antiproliferative effects in many studies *in vitro* and *in vivo* and have been therefore proposed as an auxiliary anticancer therapy in some clinical trials [39]. Recently, we showed that *INSR* gene transcription and protein expression were reduced in cells with forced expression of PPAR*γ* or TZD-induced PPAR*γ* activation (Figure 2(b)). These findings were confirmed in MCF-7 human breast cancer cells overexpressing PPAR*γ*, and 3T3-L1 adipocytes producing relatively high amounts of endogenous PPAR*γ* [40, 41]. Molecular biology studies using GST pull-down, combined with electrophoresis mobility shift assay and chromatin immunoprecipitation, have demonstrated that, in selected cell lines, PPAR*γ* physically interacts with Sp1, AP2-*α*, and C/EBP*β*, preventing binding of AP2-*α* to Sp1, as well as binding of Sp1 and C/EBP*β* to their DNA consensus sites within the *INSR* gene locus [42]. Therefore, it has been postulated that PPAR*γ* may perturb *INSR* gene expression by interfering with the transcriptional initiation complex during activation of the *INSR* gene. This observation might contribute to the identification of new therapeutic targets for treatment of tumours in which abnormal expression and/or function of INSR occur.

The INSR can be regulated by a wide variety of factors and under different environmental conditions [43]. For example, glucocorticoids enhance transcription of the *INSR* gene, whereas insulin downregulates its own receptor. As a step toward understanding the molecular basis of regulation of *INSR* gene expression, the promoter region of the human *INSR* gene has been first identified and then analyzed by several groups [44–46]. This region extends over 1800 bases upstream from the *INSR* gene ATG codon and is extremely GCrich, containing a series of GGGCGG repeats that are putative binding sites for the mammalian transcription factor Sp1. It has neither a TATA box nor a CAAT box, reflecting the common features for the promoters of constitutively expressed genes (so-called housekeeping genes). The INSR is expressed at higher levels in differentiated target tissues such as muscle and fat. At these levels, tissue-specific and ubiquitous nuclear transcription factors cooperate to induce *INSR* gene transcription. We have previously identified two distinct, functionally active DNA sequences, C2 and E3, within the *INSR* gene promoter, which had a significant ability to drive *INSR* gene transcription [46]. The molecular mechanisms regulating *INSR* gene expression have been widely studied by our group and evidence has been provided showing that the architectural transcription factor high-mobility group A1 (HMGA1) is required for proper transcription of the *INSR* gene. HMGA1 is a small basic protein that binds to AT-rich regions of certain gene promoters and functions mainly as a specific cofactor for gene activation [47–49]. HMGA1 by itself has no intrinsic transcriptional activity; rather, it has been shown to transactivate promoters through mechanisms that facilitate the assembly and stability of stereospecific DNA-protein complexes, “enhanceosomes,” that drive gene transcription. HMGA1 performs this task by modifying DNA conformation and by recruiting transcription factors to the transcription start site, facilitating DNA-protein and protein-protein interactions [47–49]. By potentiating the recruitment and binding of Sp1 and C/EBP*β* to the *INSR* promoter sequence, HMGA1 greatly enhances the transcriptional activities of these factors in this gene context [46, 50, 51]. Qualitative and/or quantitative defects in these binding proteins and/or abnormalities in their consensus sequences within the *INSR* gene may affect *INSR* gene transcription, leading to abnormalities in *INSR* gene and protein expression [26]. Overexpression of INSR in cells which normally express low levels of INSR, like epithelial cells, may increase the biological responses to insulin and trigger a ligand-mediated neoplastic transformation. Various studies have shown that INSRs are increased in most human breast cancers, and both ligand-dependent malignant transformation and increased cell growth occur in cultured breast cells overexpressing the INSR [37, 52, 53]. Also, overexpression of functional INSRs has been involved in thyroid carcinogenesis [54]. In all these cases, the INSR can exert its oncogenic potential in malignant cells via abnormal stimulation of multiple cellular signaling cascades, enhancing growth factor-dependent proliferation and/or by directly affecting cell metabolism.

## 3. Proposed Mechanisms for Hormone-Mediated Tumorigenesis

Chronic hyperinsulinemia in insulin-resistant patients increases bioavailability of IGF-I by reducing hepatic gene expression and protein production of IGFBP-1 and IGFBP-2. Also, a decrease in circulating levels of SHBG, followed by an increase in the bioavailability of estradiol and testosterone, may occur in these patients, in whom the combined effect of increased synthesis and bioavailability of estradiol and testosterone can have an adverse impact on target cells and tissues expressing estrogen and androgen receptors. The effect of sex steroid binding to their specific receptors can vary, depending on tissue type, but in some tissues (e.g., breast epithelium and endometrium), this hormone-receptor interaction results in abnormal cellular proliferation and inhibition of apoptosis. Of major importance in hormone-mediated cancers is the IGF system. This system is composed of at least three ligands (insulin, IGF-I, and IGF-II), two receptors (IGF-IR and INSR) and six structurally similar IGFBPs that have important influence over the biological effectiveness of the IGFs, since they are able to increase the half-lives of circulating IGFs, hence controlling their availability for receptor binding [55]. IGFBP-3 is the predominant binding protein expressed in serum, and the vast majority of circulating IGF-I and IGF-II are bound in a ternary complex with IGFBP-3 and a third component, the acid-labile subunit. In addition, IGFBP-3 directly regulates the interaction of IGF-I with its receptor and, through IGF-independent mechanisms, is able to inhibit cell growth and induce apoptosis. The primary location for IGFBP-3 production is in the liver, where its expression is upregulated by the growth hormone (GH) and suppressed by insulin. Because of the IGF-I's mitotic properties, lower levels of IGFBP-3, by increasing the IGF-I/IGFBP-3 ratio, may increase the risk of developing cancer, with the opposite occurring when tissue availability of IGF-I is reduced. Like IGFBP-3, the biosynthesis of IGF-I occurs primarily in the liver, where its production is GH dependent [56–58], and is increased by insulin [56, 57]. Low insulin levels, as encountered in individuals with type 1 diabetes, or following a prolonged fasting state, by determining the reduction of GH receptor expression, can contribute to lowering the hepatic IGF-I protein synthesis, thus reducing circulating levels of IGF-I. The reduced bioavailability of IGF-I under these conditions is accompanied by an increase in circulating levels of IGFBP-1 and IGFBP-2, the expression of both of which is normally suppressed by insulin. Consistently, higher expression of GH receptors with increased IGF-I protein production can be detected in patients with sustained hyperinsulinemia and T2D [59]. On the other hand, less IGFBP expression following malignant transformation has been reported in some tumour cell types in which the amount of free IGF-I may, therefore, increase even if there is no change in the rate of IGF-I production [60].

**Figure 3:**
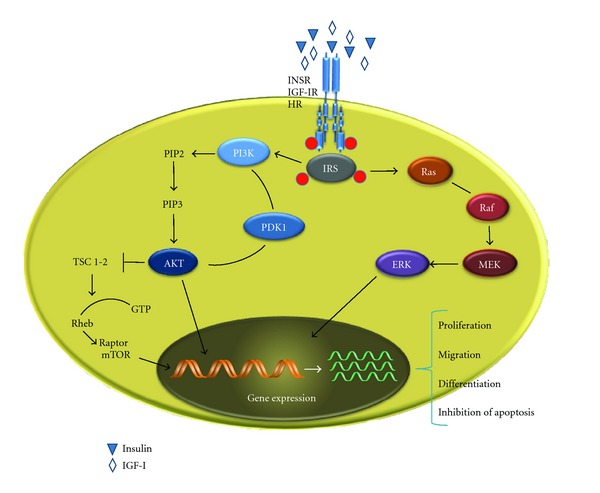
Schematic representation of the two major signaling cascades operating in cancer, following overactivation of the INSR/IGF-IR signaling pathways. Binding of insulin, IGF-I (and IGF-II) triggers the intrinsic tyrosine kinase receptor domain, leading to activation of the PI3K/Akt/mTOR signaling and the MAP/ERK-kinase pathway. HR: hybrid receptors; ERK: extracellular regulated kinase; IRS: INSR substrate; MEK: mitogen-activated protein kinase kinase; mTOR: mammalian target of rapamycin; PI3K: Phosphoinositide-3 kinase; PIP2: phosphatidylinositol [4,5]-bisphosphate; PIP3: phosphatidylinositol [3,4, 5]-trisphosphate; PDK1: phosphoinositide-dependent kinase 1; Raf: rapidly fibrosarcoma; Ras: rat sarcoma; Rheb: Ras homolog enriched in brain; TSC: tuberous sclerosis complex.

The IGF-IR is homologous to the INSR (sharing 84% amino acid identity in the intracellular tyrosine kinase domains). Because of their high sequence similarity [61, 62], an INSR hemireceptor may assemble with an IGF-IR hemireceptor, forming INSR/IGF-IR hybrid receptors. It has been demonstrated that signaling through these receptors regulates cell survival and proliferation [63, 64]. Both insulin and IGF-I bind to the extracellular *α* subunits of their cognate receptors and induce conformational changes that cause the activation of the tyrosine kinase domain and self-phosphorylation of tyrosine residues of the intracellular *β* subunit [65]. The INSR, the IGF-IR, as well as the hybrid receptors, are expressed at higher levels in malignant cells [66]. Functional activation of these receptors results in the upregulation of the INSR substrate 1 (IRS1), that triggers signaling pathways downstream of the mitogenic-activated protein (MAP) kinase pathway and the phosphoinositide-3 kinase/Akt (PI3K/Akt), two of the most important signaling cascades frequently dysregulated in cancer (Figure 3). PI3K is recruited to the membrane after being activated by growth factors and cytokines. At this level, the enzyme is activated and transfers a phosphate group to its substrate, phosphatidylinositol [4, 5]-bisphosphate [PtdIns(4,5)*P*2], forming PtdIns-(3,4,5)-*P*3 [67]. The PtdIns(3,4,5)*P*3 recruits the protein kinase Akt, facilitating its activation by the phosphoinositide-dependent kinase-1, PDK1. Phosphorylation of Akt is critical for the regulation of glucose metabolism, but also for the regulation of cell size, proliferation, and cell survival. In addition, Akt regulates gene transcription by direct phosphorylation of some of the forkhead transcription factors of the FOXO family which are involved in the control of fundamental processes, including cell metabolism and differentiation, apoptosis, cell cycle arrest, and DNA repair [68, 69]. Akt also regulates mRNA translation through the raptor-mTOR pathway, which plays a central role in metabolism and cell growth [70, 71]. The mechanism how activation of the INSR signaling pathway induces growth has been clarified by demonstrating that Akt phosphorylates and inactivates tuberin, an inhibitor of cell growth [72]. It has been shown that activation of PI3K by insulin relieves this inhibitory function [73], resulting in activation of Rheb (Ras homolog enriched in brain), leading to activation of the raptor-mTOR complex. It is well known that PTEN, a lipid phosphatase that dephosphorylates PtdIns(3,4,5)*P*3, negatively regulates the PI3K/Akt signaling pathway, thus emphasizing the role of PTEN as a tumour suppressor in multiple tumour types [74]. In this respect, PTEN is often disrupted in tumour cells, and this emphasizes the role of the insulin/IGF-I-induced PI3K/Akt/mTOR/S6K signaling in cancer [75] (Figure 3).

A second major intracellular signaling pathway involves the Ras protein, a monomeric globular protein of 189 amino acids (21 kDa) which is associated with the plasma membrane and which binds either GDP or GTP. In response to certain growth promoting stimuli, Ras is “switched on” by exchanging its bound GDP for a GTP. Once activated, Ras is able to interact with and activate other downstream protein targets. Switching Ras off requires extrinsic proteins termed GTPase-activating proteins (GAPs) that interact with Ras leading to the conversion of GTP to GDP. Mutations in Ras affecting its ability to interact with GAP, or to convert GTP to GDP, will result in abnormal, prolonged activation of this protein, thus in a sustained signal to the cell that may result in uncontrolled proliferation and disorganized growth of cells. In its active state, Ras binds Raf, a protein kinase, and promotes the activation of a phosphorylation cascade in which a series of serine/threonine protein kinases (the MAP/ERK kinase cascade) are activated in sequence, carrying the signal from the plasma membrane to the nucleus. At the end of this signal cascade, the MAP/ERK-kinase phosphorylates a number of substrates on serines and threonines, including c-Jun, c-Fos, c-Myc, Elk-1, ATF-2, NF-IL6, and TAL-1 p53, thereby modifying their ability to regulate the transcription of genes potentially relevant to cell survival, growth, and cell cycle, such as *Sp1*, *E2F*, *Elk-1,* and *AP-1* [76–79] (Figure 3).

On the whole, dysregulation of the IGF system is well recognized as an important contributor to the progression of multiple cancers, in which constitutive activation of the PI3K/Akt/mTOR signaling and the MAP/ERK-kinase pathway may play a role. Therefore, as underlined elsewhere [80], consistently with these observations, the IGF system is emerging as a promising new target in cancer therapy.

## 4. Obesity, Diabetes, and Cancer

Many clinical and epidemiological lines of evidence prove that excess body weight gain, associated with hyperinsulinemia, insulin resistance, and dyslipidemia, may be a major risk factor for certain types of tumours, including colon and breast cancer (Table 1). As illustrated in Figure 1, in this paper, besides its importance in storage and energy balance, the adipose tissue is metabolically and immunologically active, being able to produce many proteins and hormones known as “adipokines” [97], which include adipocytokines (leptin, adiponectin, and resistin), cytokines (TNF-*α*, IL-1 and IL-6), and the chemokine MCP-1 [98] that has recently been identified as a potential factor contributing to macrophage infiltration into adipose tissue [99]. Adipokines circulate in the plasma at concentrations that are positively correlated with body mass index (BMI), with the exception of adiponectin, that correlates inversely with BMI [100]. It has been demonstrated that adipocyte-secreted factors can directly promote mammary tumorigenesis through induction of antiapoptotic transcriptional programs and protooncogene stabilization [101]. Also, evidence has been provided indicating that adipocytes in obesity, by the action of adipokines, participate in a highly complex cross-talk with the surrounding tumour cells, promoting tumour progression [102]. Biosynthesis of leptin in adipose tissue is influenced by insulin [103], and this may explain the high leptin levels observed in obesity. Studies have been provided indicating that higher leptin concentrations may constitute a possible link relating obesity and cancer, particularly colorectal cancer. Also, it has been demonstrated that, by influencing specific second intracellular messengers, such as signal transducers and activators of transcription 3 (STAT3), AP-1, ERK2, and MAPK, leptin is involved in breast cancer cell proliferation and survival. On the other hand, greater adiposity in obese or overweight persons downregulates secretion of adiponectin, an adipokine with anti-inflammatory and insulin-sensitizing properties [104]. Low blood concentrations of adiponectin have been associated with high incidence and poor prognosis of breast cancer, independently from the hormone receptor status [105]. Adiponectin and adiponectin receptors have been found to play a role in the activation of the PPAR*γ* pathway, which, in turn, induces the transcription of several genes involved in the regulation of cell proliferation and differentiation. Enhancement of BRCA1 expression by PPAR*γ* has been reported in MCF-7 breast cancer cells [106]. Thus, an explanation for the association of adiponectin with breast cancer is that functional reduction of PPAR*γ* signalling, leading to reduced levels of BRCA1, may impair the DNA repair mechanisms.

Obesity and T2D are frequently associated with increased oxidative stress [107]. However, the functional role of oxidative stress in cancer has long been a hotly debated topic. Recent findings in this context indicate that oxidative stress may directly contribute to tumour progression and metastasis [108]. As recapitulated in Figure 1, one possibility is that ROS overproduction, by triggering the P13K/Akt signaling, could lead to adverse genetic modifications and DNA damage followed by tumour formation and progression [109]. NF*κ*B is a central coordinator of immunity, inflammation, and cell survival. Mutual cross-talk between ROS and NF*κ*B has been identified [110]. For example, fibroblasts harboring activated NF*κ*B are able to promote tumour growth [111]. Activation of NF*κ*B in fibroblasts leads to a loss of Cav-1 which drives onset of “The Reverse Warburg Effect,” due to the autophagic destruction of mitochondria (mitophagy) in these cells, resulting in aerobic glycolysis and lactate production [111]. Thus, by using oxidative stress, cancer cells induce the activation of the autophagic program to promote aerobic glycolysis under conditions of normoxia [111]. Therefore, treatment with antioxidants (such as N-acetyl-cysteine, metformin, quercetin, vitamins A, C, and E, selenium and perhaps others) or nitric oxide inhibitors may be beneficial to reverse many of the cancer-associated fibroblast phenotypes [112].

## 5. Inflammatory Cytokines, Diabetes, and Cancer Risk

Chronic inflammation may represent a link between diabetes and cancer, particularly in the obese, in which visceral fat is infiltrated by macrophages which constitute an important source of proinflammatory mediators [113, 114]. Moreover, macrophage accumulation in adipose tissue is associated with local hypoxia in fat [115]. It has been postulated that hypoxia in the fat tissue of the obese plays a role in the activation of inflammatory macrophages. Colocalization/coordination between macrophages/adipocytes and other cells of the immune system in white fat tissue leads to a low-grade, chronic inflammation that produces many cytokines able to initiate, promote, and sustain tumour progression either directly [116], or indirectly, by causing (via inhibition of the INSR signaling) insulin resistance, which leads to the activation of protumorigenic pathways (see Figure 1). For example, TNF-*α*, a cytokine involved in systemic inflammation, blocks insulin signaling by preventing serine phosphorylation of IRS-1 [117]. Increased expression of TNF-*α* has been observed in both acute and chronic inflammatory states, including the chronic inflammatory response associated with cancer, obesity, and diabetes. Overproduction of TNF-*α* supports and even amplifies the inflammatory process leading to insulin resistance [118]. TNF-*α* may activate both proapoptotic and antiapoptotic pathways. Under certain circumstances TNF-*α* may act as a tumour promoter by activating signaling pathways that are critical for life/death decisions, such as MAPKs and the antiapoptotic NF*κ*B pathway. Thus, increased levels of circulating TNF-*α* may promote tumorigenesis in overweight insulin-resistant patients.

Another well-characterized inflammatory cytokine, IL-6, has also been involved in various metabolic, endocrine, and neoplastic disorders. Activation of STAT signaling, via IL-6, is known to induce cancer cell proliferation, survival, and invasion, while suppressing host antitumour immunity [119]. It has been documented that the expression of IL-6 in adipose tissue and its serum concentrations positively correlate with obesity, insulin resistance, and T2D, even with insulin resistance in cancer patients [97, 120]. In one study with breast cancer patients, IL-6 and estrogen levels were found to be higher in the insulin-resistant breast cancer patients without treatment compared to the ones without insulin resistance [121]. Similarly, in prostate cancer, serum levels of IL-6 were higher in patients with obesity/insulin resistance and clinically evident hormone-resistant prostate cancer, compared to those with hormone-dependent cancer [122]. Inflammation and insulin resistance shift the cell's response to the inflammatory activating NF*κ*B, which is strongly associated with abdominal obesity and insulin resistance. As stated above, this transcription factor is involved in cytokine signaling and in cell survival, and its expression is induced by a multitude of different extracellular stimuli, including chemotherapeutics, stress stimuli, and growth factors. NF*κ*B promotes the expression of target genes involved in cellular proliferation and cell migration, anti-apoptosis, and angiogenesis. Functional reduction of NF*κ*B correlates with decreased breast tumour cell proliferation. Another mechanism that fuel cancer growth and tumour progression in low-grade chronic inflammation and insulin resistance is the accumulation of damaged DNA [123, 124]. Hyperglycemia in insulin resistance increases advanced glycation end-product (AGE) formation [125]. The production of intracellular AGE precursors damages target cells by modifying proteins and altering their function. It has been reported that plasma proteins modified by AGE precursors bind to AGE receptors on endothelial and mesangial cells and macrophages, inducing receptor-mediated production of ROS. Also, AGE receptor ligation, by activating NF*κ*B, can induce adverse changes in gene expression [126].

## 6. Conclusions

The last decades of medical research examining the pathogenesis of common tumours have provided compelling evidence for the involvement of insulin resistance in cancer. Consequently, many research articles have been published in the literature which give support to the hypothesis that patients with insulin-resistant syndromes, such as obesity and T2D, might be at higher risk for developing cancer than the general population. The molecular mechanisms for this association are unknown, but chronic sustained hyperinsulinaemia in these insulin-resistant patients appears to play a role in the neoplastic transformation process. As underlined in this paper, several explanations have been proposed for this association; however the precise mechanisms that link insulin resistance and cancer have not yet been fully understood and a more detailed molecular and mechanistic understanding is required to interpret the existing data, together with more thorough preclinical and clinical studies. Understanding these mechanisms may lead to novel diagnostic and therapeutic strategies in these patients in which measures to decrease chronic hyperinsulinemia and insulin resistance may offer a general approach to prevention of cancer.

## Figures and Tables

**Table 1:** Relative risk of association between T2D and cancer, as reported by meta-analysis studies.

Cancer	Number (*n*) of examined studies	Relative risk (CI 95%)	Reference number
Liver	Case control (*n* = 13)	2.50 (1.80–3.50)	[81]
	Cohort (*n* = 7)	2.51 (1.90–3.20)	[81]
	Cohort (*n* = 18)	2.01 (1.61–2.51)	[82]

Endometrium	Case-control (*n* = 13)	2.22 (1.80–2.74)	[83]
	Cohort (*n* = 3)	1.62 (1.21–2.16)	[83]

Pancreas	Case-control (*n* = 17)	1.94 (1.53–2.36)	[84]
	Cohort (*n* = 19)	1.73 (1.59–1.88)	[84]
	Case-control (*n* = 3)	1.80 (1.50–2.10	[85]
	Cohort (*n* = 35)	1.94 (1.66–2.27)	[86]

Kidney	Cohort (*n* = 9)	1.42 (1.06–1.91)	[87]

Biliary tract	Case-control (*n* = 8) and cohort (*n* = 13)	1.43 (1.18–1.72)	[88]
	Case-control (*n* = 10) and cohort (*n* = 5)	1.60 (1.38–1.87)	[89]

Bladder	Case-control (*n* = 7)	1.37 (1.04–1.80)	[90]
	Cohort (*n* = 3)	1.43 (1.18–1.74)	[90]

Colon-rectum	Case-control (*n* = 6)	1.36 (1.23–1.50)	[91]
	Cohort (*n* = 9)	1.29 (1.16–1.43)	[91]
	Case-control + cohort (*n* = 14)	1.38 (1.26–1.51)	[92]

Esophagus	Case-control (*n* = 6) and cohort (*n* = 11)	1.30 (1.12–1.50)	[93]

N-H lymphoma*	Case-control (*n* = 5)	1.12 (0.95–1.31)	[94]
	Cohort (*n* = 11)	1.41 (1.07–1.88)	[94]
	Case-control (*n* = 10)	1.18 (0.99–1.42)	[95]
	Cohort (*n* = 3)	1.79 (1.30–2.47)	[95]

Breast	Case-control (*n* = 5)	1.18 (1.05–1.32)	[96]
	Cohort (*n* = 15)	1.20 (1.11–1.30)	[96]

*Non-Hodgkin's lymphoma.

## Abbreviations

AGE: Advanced glycation end-product
AP2-*α*: Activator protein 2-alpha
ATF-2: Activating transcription factor-2
C/EBP*β*: CCAAT/enhancer binding protein beta
Cav-1: Caveolin-1
ERK: Extracellular regulated kinase
GAP: GTPase-activating protein
HMGA1: High-mobility group A1
IGF-I: Insulin-like growth factor-I
IGF-IR: IGF-I receptor
IGFBP: Insulin-like growth factor binding protein
IL-1: Interleukin-1
INSR: Insulin receptor
IRS-1: Insulin receptor substrate-1
MAP: Mitogenic activated protein
MCP-1: Monocyte chemoattractant protein-1
MEK: Mitogen-activated protein kinase kinase
mTOR: Mammalian target of rapamycin
NF*κ*B: Nuclear factor kappa B
PDK1: Phosphoinositide-dependent kinase 1
PI3K: Phosphoinositide-3 kinase
PPAR: Peroxisome proliferator-activated receptor
PTEN: Phosphatase and tensin homolog
Raf: Rapidly fibrosarcoma
Ras: Rat sarcoma
Rheb: Ras homolog enriched in brain
ROS: Reactive oxygen species
SHBG: Sex-hormone-binding globulin
Sp1: Specificity protein 1 transcription factor
STAT: Signal transducer and activator of transcription
T2D: Type 2 diabetes mellitus
TAL-1: T-cell acute lymphocytic leukemia protein-1
TNF-*α*: Tumour necrosis factor-alpha
TSC: Tuberous sclerosis complex.
